# Evaluation of the prognosis of acute subdural hematoma according to the density differences between gray and white matter

**DOI:** 10.3389/fneur.2022.1024018

**Published:** 2023-01-06

**Authors:** Zean Li, Yan Feng, Pengju Wang, Shuai Han, Kang Zhang, Chunyun Zhang, Shouyong Lu, Chuanxiang Lv, Fulei Zhu, Li Bie

**Affiliations:** ^1^Department of Neurosurgery of the First Clinical Hospital, Jilin University, Changchun, China; ^2^Department of Radiology of the First Clinical Hospital, Jilin University, Changchun, China

**Keywords:** acute subdural hematoma, densitometric analysis, prognosis, CT, CT quantitative analysis

## Abstract

**Objective:**

Acute subdural hematoma (ASDH) is a common neurological emergency, and its appearance on head-computed tomographic (CT) imaging helps guide clinical treatment. To provide a basis for clinical decision-making, we analyzed that the density difference between the gray and white matter of the CT image is associated with the prognosis of patients with ASDH.

**Methods:**

We analyzed the data of 194 patients who had ASDH as a result of closed traumatic brain injury (TBI) between 2018 and 2021. The patients were subdivided into surgical and non-surgical groups, and the non-surgical group was further subdivided into “diffused [hematoma]” and “non-diffused” groups. The control group's CT scans were normal. The 3D Slicer software was used to quantitatively analyze the density of gray and white matter depicted in the CT images.

**Results:**

Imaging evaluation showed that the median difference in density between the gray and white matter on the injured side was 4.12 HU (IQR, 3.91–4.22 HU; *p* < 0.001) and on the non-injured side was 4.07 HU (IQR, 3.90–4.19 HU; *p* < 0.001), and the hematoma needs to be surgically removed. The median density difference value of the gray and white matter on the injured side was 3.74 HU (IQR, 3.53–4.01 HU; *p* < 0.001) and on the non-injured side was 3.71 HU (IQR, 3.69–3.73 HU; *p* < 0.001), and the hematoma could diffuse in a short time.

**Conclusion:**

Quantitative analysis of the density differences in the gray and white matter of the CT images can be used to evaluate the clinical prognosis of patients with ASDH.

## Introduction

Acute subdural hematoma (ASDH) is a common neurosurgical emergency with high rates of morbidity and mortality. Its incidence in traumatic brain injury can reach 70%, it develops rapidly in acute cases and in seriously injured cases, and the mortality rate is as high as 50% (1–4). The prognosis of patients with ASDH depends mainly on whether the hematoma mass effect necessitates craniotomy, whether it diffuses into the subarachnoid space, whether it is stable and gradually absorbed, or whether it transforms into a chronic subdural hematoma (5–8). Early determination of patient prognosis is very important for formulating treatment plans. The Helsinki CT score, a non-quantitative assessment, is based on morphological changes shown on imaging; however, the scoring is susceptible to subjective factors (9, 10), and its utility is limited. Methods that can enable quick and accurate assessments of the prognosis of patients are urgently needed.

The severity of neurological injury can be assessed by quantitative analysis of changes in the densities of gray and white matter in brain tissue. The gray–white matter density ratio has been used to assess prognosis in patients with cardiac arrest (11–14). Primary brain injury is often accompanied by secondary brain injury, in ASDH, hematoma localized compression of brain tissue, ischemia and hypoxia of brain tissue, cell energy metabolism disorders, ion exchange disorders, and water passive entry into cells leading to cellular cerebral edema. This type of cerebral edema occurs mainly in gray and white matter cells, which is manifested as a reduced density of gray and white matter in brain tissue on CT images (15–17). As ischemia and hypoxia of brain tissue are the main mechanisms of secondary injury after craniocerebral trauma, this ratio, and changes in this ratio, can potentially help evaluate the prognosis of patients with ASDH.

To provide a basis for clinical evaluation and development of treatment plans, to assess prognosis, and to retrospectively analyze the relationship between gray and white matter density in brain CT images of patients with ASDH, we used three-dimensional modeling and quantitative analysis.

## Methods

### Patient selection

The study was approved by the local Institutional Review Board (IRB) (IRB2021350). The data are anonymous, and the requirement for informed consent was therefore waived. Patients with closed traumatic brain injury and those with normal head CT scans in the neurosurgical department of our hospital between 2018 and 2021 were involved in this study.

### Inclusion criteria

The case group included patients (1) with closed craniocerebral injury and ASDH, (2) aged 18–75 years and with a Glasgow Coma Scale (GCS) of < 13 points, (3) who had suffered the injury < 72 h earlier, and (4) in whom the supratentorial hematoma volume was 5–30 mL (calculated with computer modeling).

The control group included patients with native cranial CT scans that were performed at our neurosurgical department.

### Exclusion criteria

The case group included patients (1) with open craniocerebral injury, (2) with persistent unilateral mydriasis, (3) with infratentorial hematoma and brain stem hematoma, (4) with other injuries for more than 10% of multiple cerebral contusions.

The control group included patients (1) in whom the history of trauma was unclear, (2) with moderate to severe hydrocephalus or moderate to severe brain atrophy, and (3) combined with cerebral infarction.

### Patient grouping

The case group was subdivided into a surgical group and a non-surgical group. The latter was further subdivided into a group of patients in whom the hematoma resolved (the “diffused” group) and a group in whom the hematoma did not resolve (the “non-diffused” group) according to images of intracranial hematoma (the observation time was 7 days).

### Quantitative analysis of gray and white matter density

**Figure 1:**
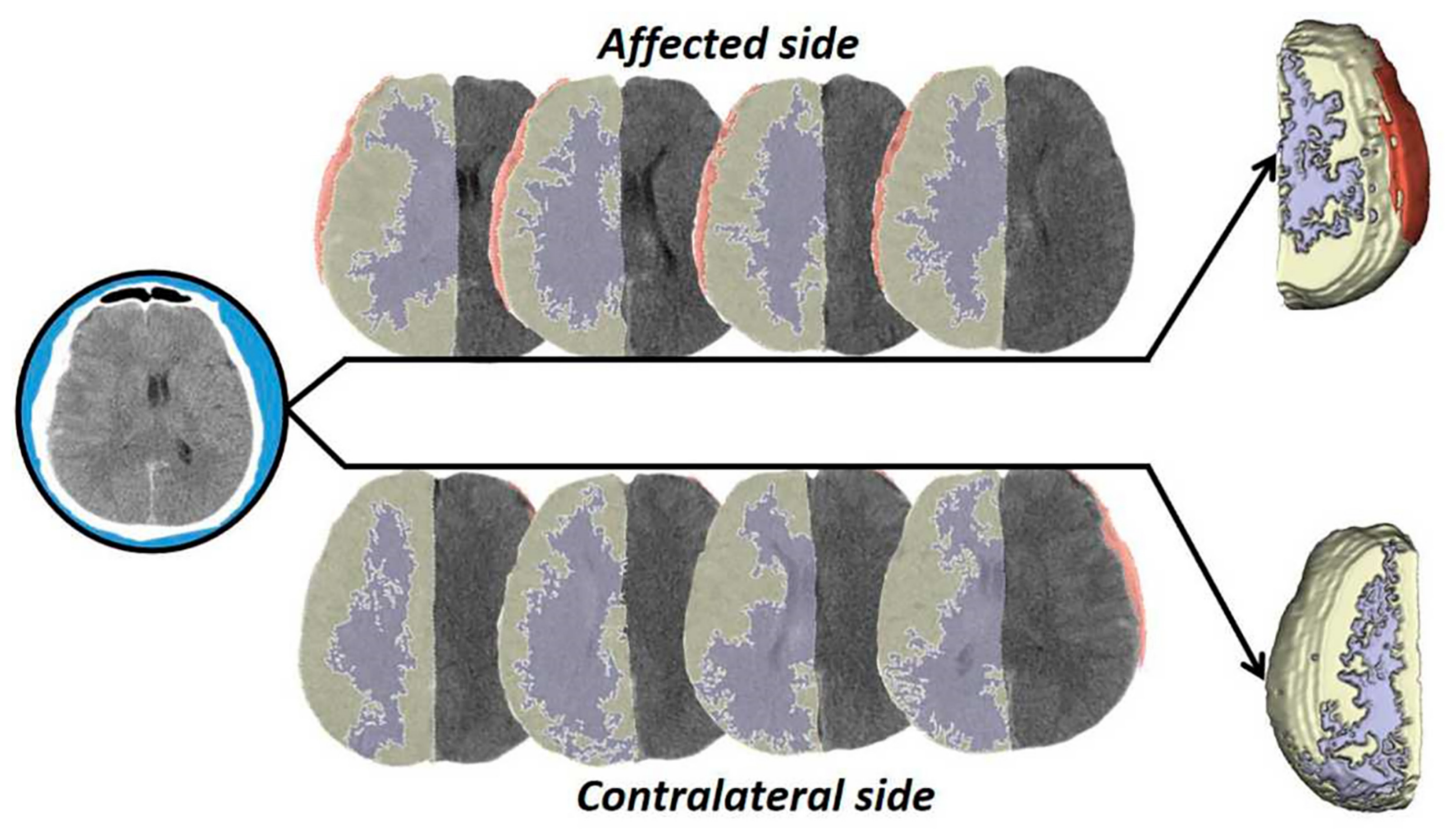
Operation flow diagram. In this study, the brain tissue CT data were processed by stratification and 3D synthesis, and the density difference was compared and analyzed.

To ensure the reliability of data analysis, the CT reviewer was blinded to the outcome. After axial CT scanning of the patient's brain tissue with a Philips Brilliance 64 CT scanner (Philips, Amsterdam, The Netherlands) with a tube voltage of 120 kV, a tube current of 250 mA, and 5-mm slices, and after exclusion of artifacts, the images of patients who met the inclusion criteria were selected for analysis. We constructed three-dimensional (3D) models of specific regions of brain tissue to be compared. The 3D Slicer version 4.10.2 software was used to analyze the density of gray and white matter for visual and quantitative analysis. In the supratentorial layer, 6–10 CT slices of the injured side were selected first, the gray matter and white matter were independently modeled, and then, the average densities and the differences between those densities were calculated. These procedures were then performed for the non-injured side. The 3D Slicer software in this study was used to customize the description of structures with similar densities and to select an area of specified ranges of density at each slice level, and then, we generated two-dimensional (2D) contours of gray matter and white matter (Figure 1). During contouring, the ventricles, skulls, intracerebral edema, and calcified plaques were erased to ensure the accuracy of post-processing data.

### Prognosis assessment

To assess long-term prognosis, we recorded Glasgow Outcome Scale–Extended (GOS-E) scores 12 months after the traumatic brain injury (TBI); 1–5 points indicated a poor prognosis, and 6–8 points indicated a good prognosis (18).

### Statistical analysis

Statistical analysis of the difference data for the gray and white matter was performed after 3D modeling. Quantitative variables were calculated as medians and interquartile ranges (IQRs), and qualitative variables were calculated as absolute frequencies and their relative percentages. One-way ANOVA, Fisher's exact test, and Dunn–Bonferroni *post-hoc* analysis were used to assess intergroup and intragroup differences. A *p-*value of < 0.05 was considered statistically significant. For comparisons of differences in gray and white matter density, we performed between-group and within-group comparisons, and we used the Bonferroni correction to control for the error rate. The statistical processing software, i.e., SPSS version 22 (IBM Corporation, Armonk, NY, USA) was used in this study.

## Results

After the initial screening of CT data, 201 patients met the inclusion criteria; artifacts appeared in the head CT scans of seven patients, and they were excluded. The study population was thus 194 patients (156 in the case group and 38 in the control group). The case group included 97 men (62.2%), whose median age was 55.3 years. Of the patients in the case group, 118 were in the non-surgical subgroup among which the hematoma diffused in 46 (38.9%) and did not in 72 (61.0%). Of the case group, 38 patients were in the surgical subgroup, of whom 17 patients (44.7%) had a GCS score of < 8 points; 17 (45.0%) had a GOS-E score >6 points 12 months after the TBI, and their prognosis was good. From all enrolled patients, we collected demographic information, physical signs at admission, history of current illness, past medical history, preoperative laboratory test results, and GOS-E score 12 months after discharge, as shown in Table 1 (Supplementary material 1).

**Table 1:** Demographic characteristics of the enrolled patients (total *n* = 156, non-surgical group *n* = 118, surgical group *n* = 38).

**Characteristic**	**Non-surgical group (*****n*** = **118)**		**Surgical group (*n* = 38)**	**Surgical (*****n*** = **38) vs. Non-surgical (*****n*** = **118)**		**Diffused (*****n*** = **46) vs. Non-diffused hematoma (*****n*** = **72)**	
	**Diffused hematoma (*n* = 46)**	**Non-diffused hematoma (*n* = 72)**		***F***	***p***	***F***	***p***
Sex (male/female)	30/16	59/13	8/30	0.003	0.955	0.620	0.540
Median age (95% CI)	53.5 [42.0, 66.0]	55 [39.5, 66.5]	57.5 [43.8, 66.3]	0.894	0.346	0.048	0.827
Mean GCS score	11.28 ± 2.67	11.23 ± 2.83	10.00 ± 4.05	2.392	0.124	0.07	0.933
**Pupil**
Mean diameter	2.96 ± 0.47	3.01 ± 0.54	3.13 ± 0.53	2.116	0.148	0.349	0.556
Light reflex, obtuse (*n*)	2 (4.34%)	4 (5.48%)	4 (10.52%)	0.074	0.786	1.448	0.231
Mean hematoma thickness (mm)	8.11 ± 2.38	8.29 ± 2.39	12.18 ± 2.73	74.371	< 0.01*	0.158	0.691
Mean midline shift (mm)	NA	NA	0.57 ± 0.10	NA	NA	NA	NA
Presence of Babinski reflex (*n*)	1	7	14	10.852	0.001*	2.542	0.114
Hypertension (*n*)	9	9	8	1.254	0.265	1.075	0.302
Diabetes (*n*)	5	3	5	1.403	0.238	1.995	0.160
Cardiopathy (*n*)	5	4	2	0.278	0.599	1.117	0.293
Liver function (*n*)	10	10	15	0.035	0.852	0.266	0.607
Median INR (IQR)	0.98 [0.93, 1.04]	1.02 [0.96, 1.065]	1.01 [0.96, 1.07]	9.276	0.003*	0.279	0.598
Median APTT (IQR)	26.1 [24.5, 28.5]	26.9 [25.1, 28.7]	28.4 [24.9, 33.2]	10.112	0.002*	1.221	0.272

APTT, activated partial thromboplastin time; CI, confidence interval; GCS, glasgow coma scale; INR, international normalized ratio; IQR, interquartile range; NA, Not applicable (Supplementary material 1). ^*^p < 0.05.

**Figure 2:**
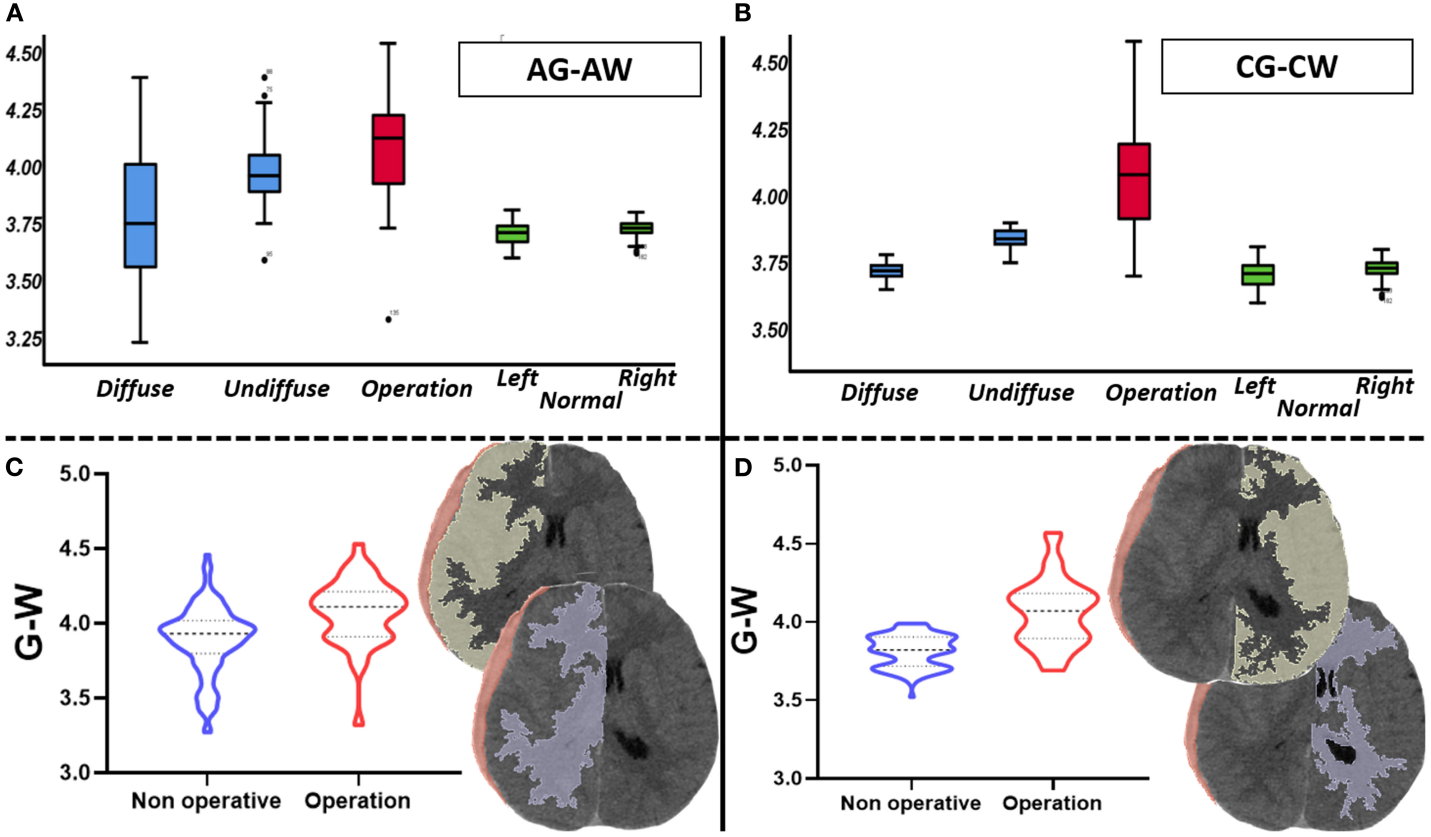
Comparison of gray and white matter's density difference value (total *n* = 156, non-surgical group *n* = 118, surgical group *n* = 38). **(A)** It mainly describes the distribution of the density differences between gray and white matter in the affected side of each group. **(B)** It mainly describes the distribution of the density differences between gray and white matter in the contralateral side of each group. **(C)** It shows the difference of the density differences between gray and white matter in affected side between surgical group and non-surgical group. **(D)** It shows the difference of the density differences between gray and white matter in contralateral side between surgical group and non-surgical group.

The quantitative analysis of 3D modeling of the gray and white matter in the injured and non-injured sides of the brain revealed that the greater the differences in gray and white matter density on the injured side, the greater the need for surgery (*p* < 0.01); if the difference was small, non-surgical treatment was preferred. In the non-surgical group, the smaller the differences in gray and white matter density on the non-injured side, the more likely the hematoma was to diffuse during treatment (*p* < 0.01) (Supplementary material 2). In comparing these differences in density between the case and control groups, we found that those differences were not statistically significant (Figure 2 and Tables 2, 3).

**Table 2:** Comparison of the differences in the density of gray and white matter in each subgroup (surgical *n* = 38, non-surgical *n* = 119, diffused hematoma *n* = 46, non-diffused hematoma *n* = 73).

**Groups**	**Affected**			**Contralateral**		
	**Median^a^**	**IQR^a^**	***p***	**Median^a^**	**IQR^a^**	***p***
Surgical vs. non-surgical	4.12	3.91–4.22	< 0.001*	4.07	3.90–4.19	< 0.001*
	3.92	3.74–4.03		3.76	3.71–3.83	
Diffused hematoma vs. non-diffused hematoma	3.74	3.53–4.01	< 0.001*	3.71	3.69–3.73	< 0.001*
	3.95	3.88–4.05		3.83	3.81–3.86	

IQR, interquartile range (Surgical n = 38 and non-surgical n = 119 and diffused hematoma n = 46 and non-diffused hematoma n = 72, Supplementary material 2).

^a^All numbers are in Hounsfield units.

^*^p < 0.05.

**Table 3:** Comparison of the density difference value between the case and control groups (disperse *n* = 119, diffused hematoma *n* = 46, non-diffused hematoma *n* = 73).

		**Normal (L)**			**Normal (R)**		
		**Median^a^**	**IQR^a^**	***p***	**Median^a^**	**IQR^a^**	***p***
Disperse (*n* = 118)	Diffused hematoma (*n* = 46)	3.74	3.53–4.01	0.211	3.71	3.69–3.73	0.240
	Non-diffused hematoma (*n* = 72)	3.70	3.66–3.74	0.331	3.72	3.70–3.74	0.616

IQR, interquartile range (Supplementary material 2).

^a^All numbers are in Hounsfield units.

With regard to coagulation dysfunction, pupillary changes, degree of midline shift, hematoma thickness, and pathological signs, we compared the risk factors associated with the prognosis of ASDH found in previous studies, and with regard to brain gray matter density, we compared the GCS score (19–22). A logistic regression analysis revealed that in the surgical group, abnormal preoperative APTT (*p* = 0.022) and differences in gray and white matter density on the injured side (*p* = 0.026) were independent risk factors for patient prognosis (Table 4 and Supplementary material 1).

**Table 4:** Multivariable analysis: OR values in surgical and non-surgical groups (surgical group *n* = 38).

**Parameter**	**OR**	**95% CI**	***p***
Presence of Babinski reflex	1.468	0.223–0.956	0.690
Median APTT, 21–33 s (IQR)	0.759	0.600–0.961	0.022
Liver function	3.304	0.466–23.397	0.231
AG–AW	1.854	1.077–3.192	0.026
CG–CW	1.275	0.800–2.030	0.307
Hematoma thickness	0.955	0.745–1.225	0.718
Midline shift	0.205	0.675–3.404	0.313

AG–AW, gray and white matter density in the injured side of the brain; APTT, activated partial thromboplastin time; CG–CW, gray and white matter densities in the non-injured side of the brain; CI, confidence ratio; OR, odds ratio (Supplementary material 3).

**Figure 3:**
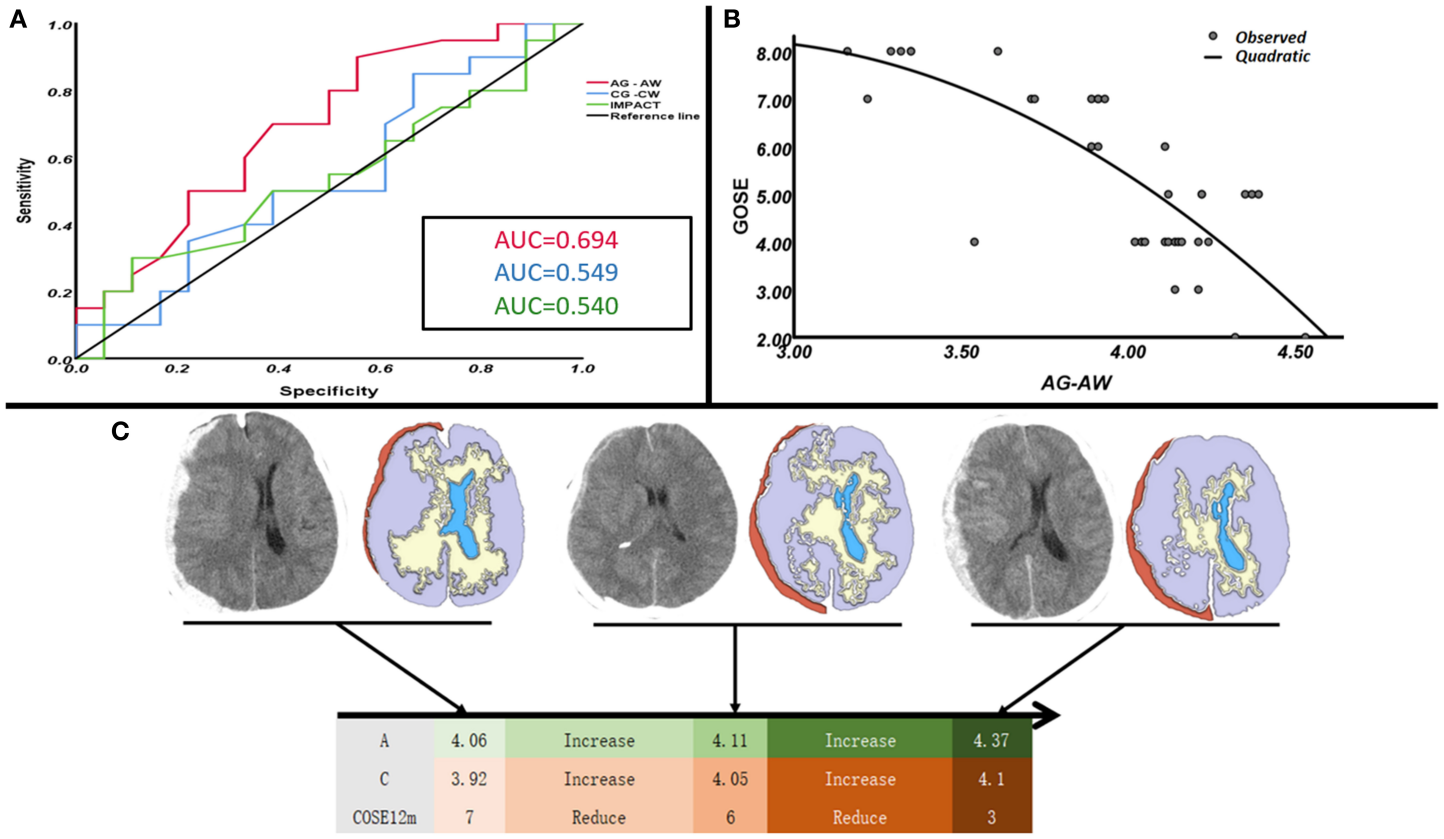
Evaluation of the prognosis of patients with ASDH using the difference between the gray matter density and the IMPACT database (surgical group *n* = 38). **(A)** To evaluate the prognosis of patients with ASDH using the gray and white matter's density difference value and the accuracy of the IMPACT database. **(B)** With the increase of the gray and white matter's density difference value, the GOSE score of patients with ASDH decreased. **(C)** In the CT visual quantitative analysis, the loss of gray and white matter density differentiation indicates a poor prognosis in ASDH patients. Note that A in **(C)**, the compressed side of the hematoma; note that C in **(C)**, the contralateral side of the hematoma. (The prognosis is assessed using the GOSE 12-month score).

Since assessing patient prognosis required a combination of clinical examination and imaging data, we used the prognostic evaluation model of the IMPACT database to improve our evaluation system. In prognostic assessment, we compared our data with those of the IMPACT database (19, 23) and calculated the corresponding areas under the curve (AUC): for the injured side, AUC = 0.750, 95% confidence ratio (CI) = 0.556–0.944; for the non-injured side, AUC = 0.609, 95% CI = 0.416–0.802; and for the IMPACT model, AUC = 0.577, 95% CI = 0.372–0.782 (Supplementary material 3). The differences in brain structures on the same side as the injury in gray and white matter density were more accurate than the IMPACT model in predicting patient prognosis, whereas in the uninjured side, the differences in gray and white matter density were similar to the IMPACT model in predicting prognosis (Figure 3). In some patients admitted to the emergency department, the volume of intracranial hematoma appeared small on CT scans, but the disease progressed rapidly, and the prognosis was poor. The 3D model of brain tissue density on the injured side in these patients showed great differences between the densities of gray and white matter. A small number of patients were admitted to the hospital on the basis of the CT appearance of a large hematoma, but their condition was actually better. From the comparison of the gray and white matter density in the 3D model of brain tissue, the difference in the density of the gray and white matter in such patients is small (Figure 3).

## Discussion

Leading to ischemia, hypoxia, and brain tissue edema, ASDH compresses brain tissue, and this mass effect results in abnormal cerebral blood flow, all of the aforementioned manifesting as abnormal gray and white matter density on CT scans (24–26). Quantitative CT analysis revealed subtle changes in gray and white matter density, and these imperceptible changes may have some correlation in the prognosis of patients with ASDH.

In comparison with the non-surgical group, the surgical group exhibited larger differences in the gray and white matter density on the injured side, mainly because the hematoma produces a mass effect, causing intracranial pressure to increase and resulting in insufficient brain perfusion and in disorders of venous return. The function of cerebral blood flow regulation, which manifests as abnormal gray and white matter density on CT images, is thereby impaired. Quantitative analysis may show that the differences in density increase, and these changes are more obvious in brain tissue that is directly compressed. Global cerebral congestion or ischemic changes and venous return obstruction after ASDH are significantly associated with a poor prognosis, which is one of the reasons why some patients with small early hematomas have a poor outcome (27–33). In the comparison between the patients with diffused hematomas and those with non-diffused hematomas, the increase in the differences in gray and white matter density in the non-injured side of the brain suggested that the hematoma does not diffuse easily in the short term. The subtle differences are reflected in the contralateral brain tissue, which exemplifies the effect of TBI on the regulation ability of the whole brain (27, 34, 35). Thus, when gray and white matter density changes, the non-injured side should not be ignored.

Multivariable analysis showed that preoperative APPT values and differences in gray matter density on the injured side (*p* < 0.05) were independent risk factors for evaluating prognosis in the surgical group. As demonstrated in our study, abnormalities of the coagulation system are associated with the progression of ASDH and poor prognosis (36–38). Brain ischemia, hypoxia, and impaired cerebral vascular regulation are closely related to a poor prognosis in patients with ASDH (39–41), and CT images can indirectly reflect the pathophysiological changes in brain tissue.

To evaluate TBI appropriately, it is necessary to account for both clinical and imaging manifestations and to comprehensively analyze and formulate treatment plans. The IMPACT database prognostic assessment model can help determine the prognosis of patients with TBI on the basis of clinical manifestations (19, 42), but the lack of support from imaging data limits the model's accuracy. The combination of clinical and imaging data enables a better assessment of the actual clinical situation. The combination of IMPACT data with CT image parameters provides the most accurate prognostic assessment of patients with ASDH, and CT image parameters enable better assessment than do the IMPACT data. The CT appearance of these pathological manifestations on the injured side of the cerebral hemisphere indicates a decrease in Hounsfield units (5, 43–48), and they are more pronounced in the injured cerebral hemisphere (Supplementary material 3).

In previous studies on differences in gray and white matter density, researchers have analyzed mainly the gray–white matter ratio using the method of planning local regions of interest. In this study, the 3D Slicer software was used to quantitatively analyze the CT images. To incorporate the post-injury changes in the whole brain into the study, we outlined the gray and white matter separately on the CT slices of the supratentorial brain tissue one by one and then performed 3D reconstruction and fusion of all slices for overall analysis. The advantage of this method is that no artificial division of brain gray matter or white matter is created, and the deep gray matter nuclei in the brain can be analyzed as well. We also evaluated the post-injury changes in the non-injured brain tissue, and thus, our evaluation was comprehensive and objective.

## Limitations

This study was a retrospective, single-center study with limited patient size. For the results of this study, a large number of prospective multicenter studies or large-scale clinical randomized controlled trials (RCT) are still needed to further validate our results.

## Conclusion

Using 3D quantitative analysis to study the changes in CT images of patients with ASDH, we found that the differences in brain gray and white matter density can indirectly reflect the pathophysiological changes in brain tissue, which are closely related to the progression of hematomas and the prognosis of patients. In our study, this method helps to determine whether a hematoma should be removed surgically on an emergency basis or whether it might resolve in a short time with conservative treatment, as shown in Figure 3. Since this study is a single-center study, the importance of this method for the development of clinical treatment plans for ASDH and the prognostic assessment of ASDH patients still needs to be verified by a large number of prospective studies.

## Data availability statement

The raw data supporting the conclusions of this article will be made available by the authors, without undue reservation.

## Ethics statement

The study was approved by the Local Institutional Review Board (IRB) (IRB2021350). Written informed consent for participation was not required for this study in accordance with the national legislation and the institutional requirements.

## Author contributions

ZL and LB contributed to study design. ZL, KZ, SH, CL, FZ, and SL contributed to data collection. ZL, YF, and PW contributed to data analysis, figures and table creation. All authors contributed to manuscript writing. All authors contributed to the article and approved the submitted version.

## Supplementary material

Supplementary material 1 Demographic characteristics of the enrolled patients (total *n* = 156, non-surgical group *n* = 118, surgical group *n* = 38).

Supplementary material 2 The density differences between gray and white matter in each group (total *n* = 156, non-surgical group *n* = 118, surgical group *n* = 38).

Supplementary material 3 Compared with IMPACT model (surgical group *n* = 38).
