# Supplementary material for: Evaluation of the prognosis of acute subdural hematoma according to the density differences between gray and white matter
Source: Front Neurol. 2023 Jan 6;13:1024018. doi: 10.3389/fneur.2022.1024018 (PMC9853902; doi:10.3389/fneur.2022.1024018)
Supplement: Supplementary material 1 — Demographic characteristics of the enrolled patients (total n = 156, non-surgical group n = 118, surgical group n = 38). [file Data_Sheet_1.PDF]

countable (n=156)	Group	Sex (Male:1/Female:2)	Age	Babinski(entity:1/nonentity:2)	Hypertension(entity:1/nonentity:2)	Diabetes(entity:1/nonentity:2)	Cardiopathy(entity:1/nonentity:2)	INR	APTT	LiverFuction(Not good:1/Good:2)	Mean diameter (Pupil)	Pupil Light reflex Sensitive:S/Obtuse:O	Mean hematoma thickness (mm)	Mean midline shift (mm)	GOSE	GCS
1	Diffused Hematoma	2	51	2	2	2	2	0.88	26	2	3	S	NA	NA	NA	13
2	Diffused Hematoma	2	66	2	1	2	2	1.08	30.8	2	2	S	NA	NA	NA	7
3	Diffused Hematoma	2	43	2	2	2	2	0.94	24.5	1	3	S	NA	NA	NA	15
4	Diffused Hematoma	1	42	2	2	2	2	0.95	24.7	2	3	S	NA	NA	NA	15
5	Diffused Hematoma	2	70	2	2	2	2	0.86	28.4	2	3	S	NA	NA	NA	15
6	Diffused Hematoma	1	56	2	1	1	1	0.93	29.5	2	3	O	NA	NA	NA	14
7	Diffused Hematoma	1	61	2	2	2	2	1.01	25	2	3	S	NA	NA	NA	15
8	Diffused Hematoma	2	66	2	2	2	2	0.91	25.5	2	3	S	NA	NA	NA	15
9	Diffused Hematoma	1	34	2	2	2	2	0.98	27.8	1	3	S	NA	NA	NA	15
10	Diffused Hematoma	2	55	2	2	2	2	0.93	25.1	2	3	S	NA	NA	NA	15
11	Diffused Hematoma	1	48	2	1	2	2	0.98	24.3	2	3	S	NA	NA	NA	13
12	Diffused Hematoma	1	52	2	2	2	2	0.9	23.7	2	3	S	NA	NA	NA	14
13	Diffused Hematoma	2	33	2	2	2	2	1.04	26.7	2	3	S	NA	NA	NA	10
14	Diffused Hematoma	1	41	2	2	2	2	0.98	27.9	1	3	S	NA	NA	NA	15
15	Diffused Hematoma	2	75	2	2	2	2	1.05	30.1	2	3	S	NA	NA	NA	15
16	Diffused Hematoma	2	52	2	2	2	2	1.08	24.7	2	3	S	NA	NA	NA	14
17	Diffused Hematoma	1	73	2	2	2	2	1.04	28.8	2	3	S	NA	NA	NA	15
18	Diffused Hematoma	1	49	2	2	2	2	0.96	25.8	2	3	S	NA	NA	NA	12
19	Diffused Hematoma	1	53	2	2	2	2	0.95	22.8	2	3	S	NA	NA	NA	15
20	Diffused Hematoma	1	31	2	2	2	2	1	31.9	2	3	S	NA	NA	NA	15
21	Diffused Hematoma	1	25	2	2	2	2	0.87	22.7	2	3	S	NA	NA	NA	11
22	Diffused Hematoma	2	53	1	1	2	2	1.11	37	2	5	S	NA	NA	NA	13
23	Diffused Hematoma	1	61	2	2	2	2	1.04	23.4	1	2	S	NA	NA	NA	14
24	Diffused Hematoma	1	31	2	2	2	2	1.02	32	1	2	S	NA	NA	NA	14
25	Diffused Hematoma	2	71	2	2	2	2	1.11	28.9	1	3	S	NA	NA	NA	15
26	Diffused Hematoma	2	68	2	1	1	1	0.89	27.2	1	3	S	NA	NA	NA	15
27	Diffused Hematoma	2	70	2	2	2	2	0.97	25.1	2	2	O	NA	NA	NA	10
28	Diffused Hematoma	2	72	2	1	2	2	0.95	27.9	1	3	S	NA	NA	NA	11
29	Diffused Hematoma	1	42	2	2	2	2	0.88	24.8	2	3	S	NA	NA	NA	14
30	Diffused Hematoma	1	34	2	2	1	2	1.08	28.4	1	3	S	NA	NA	NA	15
31	Diffused Hematoma	1	65	2	2	2	2	0.93	25.9	2	3	S	NA	NA	NA	15
32	Diffused Hematoma	1	69	2	1	2	1	1.05	24.5	2	3	S	NA	NA	NA	14
33	Diffused Hematoma	1	62	2	2	2	2	0.91	34.9	2	3	S	NA	NA	NA	12
34	Diffused Hematoma	1	52	2	2	2	2	0.97	30.6	2	2	S	NA	NA	NA	15
35	Diffused Hematoma	1	54	2	2	2	2	0.96	26.9	2	3	S	NA	NA	NA	14
36	Diffused Hematoma	1	34	2	2	2	2	1.03	26.2	2	3	S	NA	NA	NA	15
37	Diffused Hematoma	1	55	2	1	1	2	1.02	23.3	2	3	S	NA	NA	NA	13
38	Diffused Hematoma	1	62	2	2	2	1	0.95	27.5	2	3	S	NA	NA	NA	7
39	Diffused Hematoma	1	62	2	2	2	2	0.95	27.1	2	3	S	NA	NA	NA	13
40	Diffused Hematoma	1	72	2	2	2	2	0.88	23.7	1	3	S	NA	NA	NA	12
41	Diffused Hematoma	1	17	2	2	2	2	1.06	30.7	2	3	S	NA	NA	NA	15
42	Diffused Hematoma	1	35	2	2	2	2	0.98	25.6	2	3	S	NA	NA	NA	14
43	Diffused Hematoma	1	59	2	2	2	2	1.05	23.2	2	4	S	NA	NA	NA	10
44	Diffused Hematoma	1	48	2	2	2	2	0.87	24.6	2	3	S	NA	NA	NA	13
45	Diffused Hematoma	2	78	2	1	1	1	3.02	22.4	2	3	S	NA	NA	NA	14
46	Diffused Hematoma	1	42	2	2	2	2	0.92	22.6	2	3	S	NA	NA	NA	14
47	Nondiffused Hematoma	1	71	2	1	2	1	1.18	28.2	2	3	S	NA	NA	NA	9
48	Nondiffused Hematoma	1	23	2	2	2	2	1.03	28.5	2	3	S	NA	NA	NA	10
49	Nondiffused Hematoma	1	51	2	2	2	2	1.02	26.7	2	3	S	NA	NA	NA	10
50	Nondiffused Hematoma	1	66	2	2	1	2	1.01	30.7	2	3	S	NA	NA	NA	11
51	Nondiffused Hematoma	1	64	2	2	2	2	0.98	28.4	2	3	S	NA	NA	NA	9
52	Nondiffused Hematoma	1	72	2	2	2	2	1.14	28.4	2	3	S	NA	NA	NA	9
53	Nondiffused Hematoma	2	20	2	2	2	2	1.18	30.4	2	3	S	NA	NA	NA	13
54	Nondiffused Hematoma	2	75	2	1	2	2	0.86	22.7	1	3	S	NA	NA	NA	13
55	Nondiffused Hematoma	2	65	2	2	2	2	1.06	28.8	2	3	S	NA	NA	NA	11
56	Nondiffused Hematoma	1	45	2	2	2	2	0.93	26.9	2	3	S	NA	NA	NA	9
57	Nondiffused Hematoma	1	31	1	2	2	2	1.04	28.7	1	2	O	NA	NA	NA	11
58	Nondiffused Hematoma	1	52	2	2	2	2	0.96	21.7	1	3	S	NA	NA	NA	9
59	Nondiffused Hematoma	1	50	2	2	2	2	0.93	25.5	2	3	S	NA	NA	NA	12
60	Nondiffused Hematoma	1	66	2	1	2	2	0.96	26.1	2	3	S	NA	NA	NA	9
61	Nondiffused Hematoma	1	67	2	1	2	2	1.1	31.5	2	3	S	NA	NA	NA	10
62	Nondiffused Hematoma	1	71	2	2	2	2	1.02	22.2	1	3	S	NA	NA	NA	11
63	Nondiffused Hematoma	1	30	2	2	2	2	0.98	27.6	2	2	S	NA	NA	NA	12
64	Nondiffused Hematoma	2	49	2	2	2	1	1.04	27.2	2	3	S	NA	NA	NA	13
65	Nondiffused Hematoma	1	37	2	2	2	2	0.91	25.9	2	3	S	NA	NA	NA	13
66	Nondiffused Hematoma	2	49	2	2	2	1	0.98	26.4	2	3	S	NA	NA	NA	9
67	Nondiffused Hematoma	2	33	2	2	2	2	1.19	27.2	2	3	S	NA	NA	NA	11

68	Nondiffused Hematoma	1	65	2	2	2	2	0.92	24.8	2	3	S	NA	NA	NA	13
69	Nondiffused Hematoma	1	59	2	2	2	2	1.06	28.4	2	3	S	NA	NA	NA	11
70	Nondiffused Hematoma	1	68	2	2	2	2	0.97	27.4	2	3	S	NA	NA	NA	11
71	Nondiffused Hematoma	1	63	1	2	2	2	1.39	31.2	1	4	S	NA	NA	NA	9
72	Nondiffused Hematoma	1	60	2	2	2	2	1.02	24.3	2	3	S	NA	NA	NA	11
73	Nondiffused Hematoma	1	69	2	2	2	2	0.98	23.9	2	2	S	NA	NA	NA	11
74	Nondiffused Hematoma	1	53	2	2	2	2	1.2	27.6	2	3	S	NA	NA	NA	9
75	Nondiffused Hematoma	1	50	1	2	2	2	1.05	23.8	1	4	O	NA	NA	NA	13
76	Nondiffused Hematoma	1	45	2	2	2	2	1.04	24.3	2	3	S	NA	NA	NA	11
77	Nondiffused Hematoma	2	24	2	2	2	2	1.1	28.8	2	3	S	NA	NA	NA	11
78	Nondiffused Hematoma	1	51	2	2	2	2	0.9	29.1	2	3	S	NA	NA	NA	9
79	Nondiffused Hematoma	1	58	2	2	2	2	0.98	26.5	2	3	S	NA	NA	NA	14
80	Nondiffused Hematoma	1	64	2	2	2	2	0.92	29	2	3	S	NA	NA	NA	11
81	Nondiffused Hematoma	1	48	2	2	2	2	1.82	35.5	1	5	S	NA	NA	NA	11
82	Nondiffused Hematoma	1	53	2	1	2	2	1.42	32.7	2	6	S	NA	NA	NA	9
83	Nondiffused Hematoma	1	22	2	2	2	2	0.94	27	2	3	S	NA	NA	NA	13
84	Nondiffused Hematoma	1	65	2	2	2	2	0.99	26.2	2	3	S	NA	NA	NA	14
85	Nondiffused Hematoma	1	32	2	2	2	2	1.11	24.6	2	3	S	NA	NA	NA	13
86	Nondiffused Hematoma	1	55	2	2	2	2	1.01	26.4	2	3	S	NA	NA	NA	11
87	Nondiffused Hematoma	1	58	2	2	1	2	0.99	29.3	2	3	S	NA	NA	NA	13
88	Nondiffused Hematoma	1	68	2	2	2	2	1.07	26.2	2	3	S	NA	NA	NA	13
89	Nondiffused Hematoma	1	54	2	1	2	2	0.96	28.1	2	3	S	NA	NA	NA	11
90	Nondiffused Hematoma	1	87	1	2	2	2	1.12	26.6	2	3	S	NA	NA	NA	14
91	Nondiffused Hematoma	1	81	2	2	2	2	1.09	25.1	2	3	S	NA	NA	NA	9
92	Nondiffused Hematoma	1	37	2	2	2	2	1.03	26.9	2	3	S	NA	NA	NA	12
93	Nondiffused Hematoma	1	10	2	2	2	2	1.03	27.6	2	3	S	NA	NA	NA	11
94	Nondiffused Hematoma	1	58	2	2	2	2	0.95	27.6	2	3	S	NA	NA	NA	9
95	Nondiffused Hematoma	1	55	2	2	2	2	0.96	24	1	2	S	NA	NA	NA	15
96	Nondiffused Hematoma	1	55	2	2	2	2	1.05	28.7	2	3	S	NA	NA	NA	14
97	Nondiffused Hematoma	1	68	2	2	2	2	1.03	28.5	2	3	S	NA	NA	NA	9
98	Nondiffused Hematoma	1	67	2	2	2	2	0.92	24.9	2	2	S	NA	NA	NA	9
99	Nondiffused Hematoma	2	46	2	2	2	2	1.02	25.4	2	3	S	NA	NA	NA	14
100	Nondiffused Hematoma	2	21	2	2	2	2	1.09	25.4	2	3	S	NA	NA	NA	9
101	Nondiffused Hematoma	2	78	2	1	2	2	0.95	26.3	2	3	S	NA	NA	NA	13
102	Nondiffused Hematoma	1	83	2	2	2	2	1	27.7	2	3	S	NA	NA	NA	13
103	Nondiffused Hematoma	1	45	1	2	2	2	1.44	40.5	2	3	S	NA	NA	NA	13
104	Nondiffused Hematoma	1	32	2	2	2	2	1.14	31.7	2	3	S	NA	NA	NA	9
105	Nondiffused Hematoma	1	58	2	2	2	2	0.89	30.2	2	3	S	NA	NA	NA	13
106	Nondiffused Hematoma	1	34	2	2	2	2	0.96	28	2	3	S	NA	NA	NA	14
107	Nondiffused Hematoma	1	57	2	2	2	2	1	33	2	3	S	NA	NA	NA	15
108	Nondiffused Hematoma	1	53	2	2	2	2	1.02	23.1	2	3	S	NA	NA	NA	15
109	Nondiffused Hematoma	1	60	1	2	2	2	1.03	21	1	3	S	NA	NA	NA	9
110	Nondiffused Hematoma	1	32	2	2	2	2	0.94	17.5	2	3	S	NA	NA	NA	9
111	Nondiffused Hematoma	2	68	2	2	2	2	0.92	24	2	3	S	NA	NA	NA	13
112	Nondiffused Hematoma	1	42	2	2	2	2	0.95	24.8	2	3	S	NA	NA	NA	13
113	Nondiffused Hematoma	2	28	2	2	2	2	0.91	25.1	2	3	S	NA	NA	NA	11
114	Nondiffused Hematoma	2	80	2	1	1	1	1	24.1	2	3	S	NA	NA	NA	13
115	Nondiffused Hematoma	1	61	2	1	2	2	1.02	25.6	2	3	S	NA	NA	NA	11
116	Nondiffused Hematoma	1	33	2	2	2	2	1.05	26.5	2	3	S	NA	NA	NA	11
117	Nondiffused Hematoma	1	14	1	2	2	2	1.23	25.3	1	3	S	NA	NA	NA	13
118	Nondiffused Hematoma	1	62	2	2	2	2	0.93	30.8	2	2	S	NA	NA	NA	11
119	Surgical	1	38	2	2	2	2	1.14	30.5	1	3	S	10	0.5	4	6
120	Surgical	1	64	1	2	2	2	1.03	36	2	3	S	13	0.5	6	5
121	Surgical	1	61	2	2	2	2	1.06	29.2	2	3	S	9	0.5	5	9
122	Surgical	2	53	1	2	2	2	1.34	30.5	1	5	S	16	0.8	5	15
123	Surgical	1	75	2	1	1	1	1.32	35	1	3	S	12	0.5	5	15
124	Surgical	2	38	1	2	2	2	0.96	25.4	2	4	O	10	0.6	4	8
125	Surgical	2	57	2	2	2	2	0.98	24.8	2	3	S	17	0.5	5	7
126	Surgical	1	59	1	1	2	2	0.92	33.1	1	3	S	11	0.7	6	15
127	Surgical	1	41	1	2	2	2	0.97	30.6	2	3	S	10	0.5	6	6
128	Surgical	1	63	2	2	2	2	1.09	33.7	1	3	S	11	0.5	4	14
129	Surgical	1	24	2	2	2	2	1.01	24.3	2	3	S	16	0.6	7	8
130	Surgical	1	74	1	1	1	2	1.04	36.1	1	4	O	13	0.5	5	7
131	Surgical	1	44	2	2	2	2	0.97	24.9	1	3	S	19	0.8	6	11
132	Surgical	1	52	2	2	2	2	0.94	22.8	2	3	S	20	0.7	7	7
133	Surgical	1	34	1	2	2	2	1.13	28.9	2	3	S	9	0.5	6	10
134	Surgical	1	43	1	2	2	2	1.09	27.1	1	3	S	13	0.5	4	6
135	Surgical	2	77	2	2	2	2	0.97	29.8	2	3	S	11	0.5	4	13
136	Surgical	1	61	1	2	2	2	1.12	31.7	2	6	O	15	0.6	5	8
137	Surgical	1	42	2	2	2	2	0.96	22.6	1	3	S	11	0.5	6	11
138	Surgical	2	47	1	2	2	2	0.95	22.8	1	3	S	11	0.5	5	9
139	Surgical	1	64	2	2	2	2	0.97	30.9	2	3	S	13	0.6	6	13

140	Surgical	1	51	2	2	2	2	1.05	27.3	2	3	S	15	0.5	5	13
141	Surgical	1	39	2	2	2	2	1.05	25.1	1	3	S	10	0.5	4	13
142	Surgical	1	54	2	1	1	1	0.96	34.8	1	3	S	10	0.6	5	14
143	Surgical	1	61	2	2	2	2	1.28	39	1	6	S	10	0.7	6	6
144	Surgical	1	65	2	1	2	2	1.02	27.4	2	7	S	10	0.7	6	5
145	Surgical	1	72	2	2	2	2	1.04	27.8	1	3	S	13	0.5	4	8
146	Surgical	2	71	2	1	2	2	0.99	27.6	2	3	S	11	0.5	7	14
147	Surgical	2	68	2	1	1	2	0.96	32.9	2	3	S	11	0.6	5	15
148	Surgical	1	69	2	2	2	2	0.92	35.4	2	3	S	13	0.5	5	14
149	Surgical	1	54	2	2	2	2	1.03	33.9	2	3	S	11	0.8	6	8
150	Surgical	1	47	2	2	2	2	1.01	24.3	1	3	S	10	0.6	6	10
151	Surgical	1	79	2	1	1	2	1.12	31.5	2	3	S	11	0.7	7	14
152	Surgical	1	64	2	2	2	2	0.98	27.3	2	3	S	15	0.5	4	14
153	Surgical	1	58	2	2	2	2	1.27	33.4	1	4	S	13	0.5	5	6
154	Surgical	2	29	2	2	2	2	0.96	20.3	2	3	S	11	0.5	4	13
155	Surgical	1	66	2	2	2	2	0.91	22.1	2	3	S	10	0.6	6	8
156	Surgical	1	55	2	2	2	2	1.01	26.2	2	3	S	9	0.6	6	14
