# Supplementary material for: Evaluation of the prognosis of acute subdural hematoma according to the density differences between gray and white matter
Source: Front Neurol. 2023 Jan 6;13:1024018. doi: 10.3389/fneur.2022.1024018 (PMC9853902; doi:10.3389/fneur.2022.1024018)
Supplement: Supplementary material 2 — The density differences between gray and white matter in each group (total n = 156, non-surgical group n = 118, surgical group n = 38). [file Data_Sheet_2.PDF]

countable (n=156)	Group	AG-AW	CG-CW	countable	Normal(LG-LW)	Normal(RG-RW)
1	Diffused Hematoma	3.72	3.71	1	3.67	3.73
2	Diffused Hematoma	3.65	3.69	2	3.72	3.74
3	Diffused Hematoma	3.86	3.68	3	3.76	3.75
4	Diffused Hematoma	3.41	3.65	4	3.70	3.71
5	Diffused Hematoma	3.50	3.61	5	3.80	3.75
6	Diffused Hematoma	4.00	3.72	6	3.65	3.74
7	Diffused Hematoma	4.04	3.77	7	3.61	3.74
8	Diffused Hematoma	3.48	3.66	8	3.73	3.78
9	Diffused Hematoma	3.98	3.75	9	3.77	3.79
10	Diffused Hematoma	4.01	3.78	10	3.63	3.62
11	Diffused Hematoma	3.49	3.66	11	3.69	3.72
12	Diffused Hematoma	3.43	3.69	12	3.70	3.71
13	Diffused Hematoma	3.42	3.64	13	3.68	3.73
14	Diffused Hematoma	3.45	3.69	14	3.65	3.71
15	Diffused Hematoma	3.62	3.77	15	3.70	3.73
16	Diffused Hematoma	3.55	3.75	16	3.73	3.68
17	Diffused Hematoma	4.00	3.94	17	3.73	3.74
18	Diffused Hematoma	4.07	3.71	18	3.65	3.74
19	Diffused Hematoma	3.27	3.82	19	3.74	3.77
20	Diffused Hematoma	3.71	3.82	20	3.65	3.64
21	Diffused Hematoma	4.02	3.90	21	3.59	3.69
22	Diffused Hematoma	3.57	3.71	22	3.75	3.71
23	Diffused Hematoma	3.73	3.71	23	3.78	3.70
24	Diffused Hematoma	3.52	3.68	24	3.66	3.67
25	Diffused Hematoma	4.02	3.70	25	3.71	3.72
26	Diffused Hematoma	4.08	3.68	26	3.72	3.75
27	Diffused Hematoma	3.68	3.72	27	3.70	3.73
28	Diffused Hematoma	4.00	3.71	28	3.70	3.73
29	Diffused Hematoma	3.78	3.73	29	3.73	3.72
30	Diffused Hematoma	3.73	3.67	30	3.74	3.72
31	Diffused Hematoma	4.04	3.69	31	3.66	3.61
32	Diffused Hematoma	4.11	3.52	32	3.70	3.69
33	Diffused Hematoma	3.67	3.64	33	3.74	3.72
34	Diffused Hematoma	4.38	3.71	34	3.72	3.72
35	Diffused Hematoma	3.74	3.67	35	3.63	3.64
36	Diffused Hematoma	3.41	3.72	36	3.77	3.78
37	Diffused Hematoma	3.79	3.73	37	3.72	3.68
38	Diffused Hematoma	3.61	3.68			
39	Diffused Hematoma	4.08	3.72			
40	Diffused Hematoma	3.50	3.70			
41	Diffused Hematoma	4.09	3.72			
42	Diffused Hematoma	3.97	3.70			
43	Diffused Hematoma	4.00	3.69			
44	Diffused Hematoma	3.88	3.67			
45	Diffused Hematoma	4.04	3.77			
46	Diffused Hematoma	3.53	3.71			
47	Nondiffused Hematoma	3.94	3.84			
48	Nondiffused Hematoma	4.08	3.80			
49	Nondiffused Hematoma	3.82	3.81			
50	Nondiffused Hematoma	3.29	3.78			
51	Nondiffused Hematoma	4.30	3.84			
52	Nondiffused Hematoma	3.96	3.79			
53	Nondiffused Hematoma	3.80	3.82			
54	Nondiffused Hematoma	3.86	3.86			
55	Nondiffused Hematoma	3.92	3.90			
56	Nondiffused Hematoma	3.84	3.90			
57	Nondiffused Hematoma	3.83	3.89			
58	Nondiffused Hematoma	4.11	3.87			
59	Nondiffused Hematoma	3.75	3.98			
60	Nondiffused Hematoma	3.84	3.88			
61	Nondiffused Hematoma	3.96	3.91			
62	Nondiffused Hematoma	4.20	3.81			
63	Nondiffused Hematoma	3.82	3.88			
64	Nondiffused Hematoma	3.92	3.99			
65	Nondiffused Hematoma	3.98	3.87			
66	Nondiffused Hematoma	3.86	3.84			
67	Nondiffused Hematoma	4.13	3.84			
68	Nondiffused Hematoma	3.74	3.91			
69	Nondiffused Hematoma	3.97	3.99			
70	Nondiffused Hematoma	3.89	3.87			
71	Nondiffused Hematoma	3.96	3.93			
72	Nondiffused Hematoma	3.93	3.85			
73	Nondiffused Hematoma	3.88	3.91			
74	Nondiffused Hematoma	3.91	3.98			
75	Nondiffused Hematoma	3.84	3.87			
76	Nondiffused Hematoma	3.93	3.95			
77	Nondiffused Hematoma	4.13	3.97			
78	Nondiffused Hematoma	3.95	3.81			
79	Nondiffused Hematoma	3.87	3.86			
80	Nondiffused Hematoma	3.98	3.82			
81	Nondiffused Hematoma	4.06	3.92			
82	Nondiffused Hematoma	3.81	3.87			
83	Nondiffused Hematoma	4.15	3.97			
84	Nondiffused Hematoma	3.99	3.82			
85	Nondiffused Hematoma	3.81	3.98			
86	Nondiffused Hematoma	3.93	3.93			
87	Nondiffused Hematoma	4.17	3.93			
88	Nondiffused Hematoma	4.46	3.84			
89	Nondiffused Hematoma	3.95	3.91			
90	Nondiffused Hematoma	4.18	3.87			
91	Nondiffused Hematoma	3.87	3.81			
92	Nondiffused Hematoma	3.95	3.99			
93	Nondiffused Hematoma	4.24	3.92			
94	Nondiffused Hematoma	3.98	3.89			
95	Nondiffused Hematoma	3.88	3.91			
96	Nondiffused Hematoma	4.17	3.85			
97	Nondiffused Hematoma	3.88	3.81			
98	Nondiffused Hematoma	3.93	3.89			
99	Nondiffused Hematoma	3.89	3.81			
100	Nondiffused Hematoma	4.08	3.92			
101	Nondiffused Hematoma	3.88	3.91			
102	Nondiffused Hematoma	3.98	3.82			
103	Nondiffused Hematoma	4.15	3.92			
104	Nondiffused Hematoma	4.18	3.91			
105	Nondiffused Hematoma	3.93	3.81			

106	Nondiffused Hematoma	3.99	3.83
107	Nondiffused Hematoma	3.91	3.93
108	Nondiffused Hematoma	3.96	3.89
109	Nondiffused Hematoma	3.99	3.91
110	Nondiffused Hematoma	4.10	3.86
111	Nondiffused Hematoma	3.88	3.83
112	Nondiffused Hematoma	3.97	3.91
113	Nondiffused Hematoma	3.95	3.83
114	Nondiffused Hematoma	3.93	3.81
115	Nondiffused Hematoma	3.82	3.81
116	Nondiffused Hematoma	3.91	3.87
117	Nondiffused Hematoma	4.03	3.91
118	Nondiffused Hematoma	4.37	3.95
119	Surgical	4.37	3.95
120	Surgical	4.35	3.88
121	Surgical	4.11	3.99
122	Surgical	4.21	4.19
123	Surgical	4.22	3.92
124	Surgical	4.39	4.26
125	Surgical	3.93	3.86
126	Surgical	4.14	4.09
127	Surgical	4.06	4.10
128	Surgical	4.32	4.11
129	Surgical	4.02	4.28
130	Surgical	4.16	3.78
131	Surgical	4.29	4.20
132	Surgical	4.14	4.13
133	Surgical	4.21	4.16
134	Surgical	3.92	3.90
135	Surgical	4.24	4.12
136	Surgical	3.91	3.96
137	Surgical	4.22	3.83
138	Surgical	3.85	3.77
139	Surgical	4.11	4.05
140	Surgical	4.04	4.03
141	Surgical	4.53	4.57
142	Surgical	4.12	4.18
143	Surgical	3.91	3.70
144	Surgical	3.91	4.05
145	Surgical	4.21	4.21
146	Surgical	4.05	3.69
147	Surgical	4.12	4.16
148	Surgical	4.11	4.17
149	Surgical	3.91	3.92
150	Surgical	3.72	4.16
151	Surgical	3.89	3.84
152	Surgical	4.04	4.54
153	Surgical	3.89	4.39
154	Surgical	4.15	3.91
155	Surgical	3.75	3.88
156	Surgical	3.32	4.37
