# Supplementary material for: Evaluation of the prognosis of acute subdural hematoma according to the density differences between gray and white matter
Source: Front Neurol. 2023 Jan 6;13:1024018. doi: 10.3389/fneur.2022.1024018 (PMC9853902; doi:10.3389/fneur.2022.1024018)
Supplement: Supplementary material 3 — Compared with IMPACT model (surgical group n = 38). [file Data_Sheet_3.PDF]

| countable (n=38) | group | AG-AW | CG-CW | IMPACT | | GOSE(1 – 5 points indicated a poor prognosis, and 6–8 points indicated a good prognosis) | Babinski(entity:1/non entity:2 ) | APTT | LiverFuction(Not good:1/Good:2) | Mean hematoma thickness (mm) | Mean midline shift (mm) |
|---|---|---|---|---|---|---|---|---|---|---|---|
| 1 | Surgical | 4.37 | 3.95 | 0.4 | 0.38 | 4 | 2 | 30.5 | 1 | 10 | 0.5 |
| 2 | Surgical | 4.35 | 3.88 | 0.66 | 0.61 | 6 | 1 | 36 | 2 | 13 | 0.5 |
| 3 | Surgical | 4.11 | 3.99 | 0.63 | 0.58 | 5 | 2 | 29.2 | 2 | 9 | 0.5 |
| 4 | Surgical | 3.61 | 4.19 | 0.56 | 0.52 | 5 | 1 | 30.5 | 1 | 16 | 0.8 |
| 5 | Surgical | 4.22 | 3.92 | 0.74 | 0.69 | 5 | 2 | 35 | 1 | 12 | 0.5 |
| 6 | Surgical | 4.39 | 4.26 | 0.42 | 0.39 | 4 | 1 | 25.4 | 2 | 10 | 0.6 |
| 7 | Surgical | 3.93 | 3.86 | 0.59 | 0.55 | 5 | 2 | 24.8 | 2 | 17 | 0.5 |
| 8 | Surgical | 4.14 | 4.09 | 0.61 | 0.57 | 6 | 1 | 33.1 | 1 | 11 | 0.7 |
| 9 | Surgical | 3.16 | 4.1 | 0.45 | 0.42 | 6 | 1 | 30.6 | 2 | 10 | 0.5 |
| 10 | Surgical | 4.32 | 4.11 | 0.65 | 0.60 | 4 | 2 | 33.7 | 1 | 11 | 0.5 |
| 11 | Surgical | 4.02 | 4.28 | 0.3 | 0.29 | 7 | 2 | 24.3 | 2 | 16 | 0.6 |
| 12 | Surgical | 4.16 | 3.78 | 0.74 | 0.68 | 5 | 1 | 36.1 | 1 | 13 | 0.5 |
| 13 | Surgical | 3.29 | 4.2 | 0.47 | 0.44 | 6 | 2 | 24.9 | 1 | 19 | 0.7 |
| 14 | Surgical | 4.14 | 4.13 | 0.55 | 0.51 | 7 | 2 | 22.8 | 2 | 20 | 0.6 |
| 15 | Surgical | 4.21 | 4.16 | 0.38 | 0.36 | 6 | 1 | 28.9 | 2 | 9 | 0.5 |
| 16 | Surgical | 3.22 | 3.9 | 0.46 | 0.43 | 4 | 1 | 27.1 | 1 | 13 | 0.5 |
| 17 | Surgical | 4.24 | 4.12 | 0.76 | 0.7 | 4 | 2 | 29.8 | 2 | 11 | 0.5 |
| 18 | Surgical | 3.91 | 3.96 | 0.63 | 0.58 | 5 | 1 | 31.7 | 2 | 15 | 0.6 |
| 19 | Surgical | 4.22 | 3.83 | 0.45 | 0.43 | 6 | 2 | 22.6 | 1 | 11 | 0.5 |
| 20 | Surgical | 3.35 | 3.77 | 0.5 | 0.47 | 5 | 1 | 22.8 | 1 | 11 | 0.5 |
| 21 | Surgical | 4.11 | 4.05 | 0.66 | 0.61 | 6 | 2 | 30.9 | 2 | 13 | 0.5 |
| 22 | Surgical | 3.54 | 4.03 | 0.54 | 0.50 | 5 | 2 | 27.3 | 2 | 15 | 0.5 |
| 23 | Surgical | 4.53 | 4.57 | 0.43 | 0.40 | 4 | 2 | 25.1 | 1 | 10 | 0.5 |
| 24 | Surgical | 4.12 | 4.18 | 0.57 | 0.53 | 5 | 2 | 34.8 | 1 | 10 | 0.6 |
| 25 | Surgical | 3.91 | 3.7 | 0.63 | 0.58 | 6 | 2 | 39 | 1 | 10 | 0.6 |
| 26 | Surgical | 3.91 | 4.05 | 0.66 | 0.61 | 6 | 2 | 27.4 | 2 | 10 | 0.6 |
| 27 | Surgical | 4.21 | 4.21 | 0.72 | 0.67 | 4 | 2 | 27.8 | 1 | 13 | 0.5 |
| 28 | Surgical | 4.05 | 3.69 | 0.71 | 0.66 | 7 | 2 | 27.6 | 2 | 11 | 0.5 |
| 29 | Surgical | 4.12 | 4.16 | 0.69 | 0.64 | 5 | 2 | 32.9 | 2 | 11 | 0.6 |
| 30 | Surgical | 3.71 | 4.17 | 0.70 | 0.65 | 5 | 2 | 35.4 | 2 | 13 | 0.5 |
| 31 | Surgical | 3.91 | 3.92 | 0.57 | 0.53 | 6 | 2 | 33.9 | 2 | 11 | 0.8 |
| 32 | Surgical | 3.72 | 4.16 | 0.50 | 0.47 | 6 | 2 | 24.3 | 1 | 10 | 0.6 |
| 33 | Surgical | 3.89 | 3.84 | 0.77 | 0.72 | 7 | 2 | 31.5 | 2 | 11 | 0.7 |
| 34 | Surgical | 4.04 | 4.54 | 0.66 | 0.61 | 4 | 2 | 27.3 | 2 | 15 | 0.5 |
| 35 | Surgical | 3.89 | 4.39 | 0.60 | 0.56 | 5 | 2 | 33.4 | 1 | 13 | 0.5 |
| 36 | Surgical | 4.15 | 3.91 | 0.34 | 0.33 | 4 | 2 | 20.3 | 2 | 11 | 0.5 |
| 37 | Surgical | 3.35 | 3.88 | 0.67 | 0.62 | 6 | 2 | 22.1 | 2 | 10 | 0.6 |
| 38 | Surgical | 3.32 | 4.37 | 0.58 | 0.53 | 6 | 2 | 26.2 | 2 | 9 | 0.6 |
